# *Castanea* spp. Agrobiodiversity Conservation: Genotype Influence on Chemical and Sensorial Traits of Cultivars Grown on the Same Clonal Rootstock

**DOI:** 10.3390/foods9081062

**Published:** 2020-08-05

**Authors:** Gabriele L. Beccaro, Dario Donno, Guglielmo Gianni Lione, Marta De Biaggi, Giovanni Gamba, Sabrina Rapalino, Isidoro Riondato, Paolo Gonthier, Maria Gabriella Mellano

**Affiliations:** 1 Dipartimento di Scienze Agrarie, Forestali e Alimentari, Università degli Studi di Torino, 10124 Torino, Italy; gabriele.beccaro@unito.it (G.L.B.); guglielmo.lione@unito.it (G.G.L.); m.debiaggi@hotmail.com (M.D.B.); giovanni.gamba@unito.it (G.G.); rapalino-sabrina@hotmail.it (S.R.); isidoro.riondato@unito.it (I.R.); paolo.gonthier@unito.it (P.G.); gabriella.mellano@unito.it (M.G.M.); 2 Chestnut R&D Center—Piemonte, 12013 Chiusa di Pesio, Italy

**Keywords:** chestnut, characterisation tool, bioactive compounds, sensory analysis, multivariate approach

## Abstract

A large species diversity characterises the wide distribution of chestnuts in Asia, North America, and Europe, hence reflecting not only the adaptation of the genus *Castanea* to diverse environmental conditions, but also to different management strategies encompassing orchards. The characterisation and description of chestnut populations and cultivars are crucial to develop effective conservation strategies of one of the most important Italian and European fruit and wood species. Chestnut cultivars grown in the same pedoclimatic conditions and on the same clonal rootstock were characterised with sensory, spectrophotometric, and chromatographic analysis to determine the phytochemical composition and nutraceutical properties. A multivariate approach, including principal component analysis and conditional inference tree models, was also performed. The ease of peeling, seed colour, and intensity of sweetness were the sensory descriptors that allowed us to differentiate *C. sativa* cultivars. Antioxidant capacity ranged from 9.30 ± 0.39 mmol Fe^+2^ kg^−1^ DW (‘Bouche de Bètizac’) to 19.96 ± 1.89 mmol Fe^+2^ kg^−1^ DW (‘Garrone Rosso’). Monoterpenes represented the main component, reaching 88% for hybrids, followed by polyphenols (10–25% for hybrids and chestnuts, respectively). A multivariate approach showed that phenolic acids and tannins were the bioactive classes with the highest discriminating power among different genotypes, and that genotype is a significant variable (*p* < 0.05). In addition, most of the analysed chestnut cultivars showed a content of bioactive compounds similar to or higher than the main hazelnut, walnut, and almond varieties. Chestnut agrobiodiversity could be intended as strictly associated to the genotype effect and underlines the large variability within the genus *Castanea*, and therefore, the importance of in farm and ex situ conservation of local germplasm is part of a global strategy aimed at increasing the levels of agrobiodiversity.

## 1. Introduction

Landscape modification, habitat loss, and fragmentation stand among the main agrobiodiversity conservation issues that affect the ecosystem structure and functioning with negative effects on plants populations and communities [1]. The consequences of these pressures is particularly true for species with a relevant environmental and historical economic role in agroforestry systems, such as chestnut (*Castanea* spp.) [2].

A large species diversity characterises the wide distribution of chestnut in Asia, North America, and Europe, hence reflecting not only the adaptation of the genus *Castanea* to diverse environmental conditions, but also to different management strategies encompassing orchards for fruit production, coppices for timber production, and naturalised populations providing several ecosystem services [3]. Indeed, the high levels of chestnut diversity are largely acknowledged as the result of the co-existence between *Castanea* spp. and human populations [4].

Natural and planted forests of sweet chestnut cover the species’ ecological limits [5], spreading from the Caucasus to Portugal, reaching the southern United Kingdom, Canary Islands, and the Azores archipelago. It is also locally present in Lebanon and Syria [6]. Sweet chestnut is one of the oldest domesticated species, widespread throughout the Roman Empire and commonly cultivated during the Medieval period, becoming so indispensable for the survival of mountain populations that these cultures were identified as “chestnut civilizations” [7]. Therefore, sweet chestnut represents an important resource in Europe for its great ecological (large ecosystem biodiversity and landscape value), economic (fruit, wood, honey, and tannin production), and cultural relevance. Noteworthily, sweet chestnut is considered one of the most important trees in Italy [8], underlined by the presence of ancient forests and orchards with monumental trees [2,9].

However, since the beginning of the 20th century, the growing areas of sweet chestnut have dramatically decreased because of several social, cultural, and environmental changes. Such challenges include the progressive depopulation of mountain areas, diet changes [10,11], climate change [12], and the establishment and spread of diseases and pests. The latter encompass ink disease caused by the oomycetes *Phytophthora cambivora* (Petri) Buisman and *P. cinnamomi* Rands, chestnut blight associated with the ascomycete *Cryphonectria parasitica* (Murrill) M.E. Barr, the emerging nut rot due to *Gnomoniopsis castaneae* G. Tamietti, and the infestation and control of the Asian gall wasp *Dryocosmus kuriphilus* Yasumatsu [13,14,15,16]. The preservation of chestnut intra- and interspecific diversity together with a management hinging on regular agronomic treatments seems to be the key factors for a correct management of the plantations [17]. The above diversity can also contribute to the selection of varieties more tolerant or resistant to diseases and pests, and not surprisingly, breeding programs and preservation strategies for the existing varieties are sought, implemented, and supported worldwide [18]. Moreover, genetic and ecophysiological investigations on *C. sativa* showed the wide plasticity of the species to cope with different environments (water and nutrient uptake efficiency) [19].

For these reasons, the characterisation and description of chestnut populations and cultivars [20] are crucial to develop effective conservation strategies of one of the most important Italian and European fruit and wood species [2,17]. A genetic landscape study on the European sweet chestnut was recently performed to evaluate the geographical patterns of its diversity and to identify high-priority areas characterised by high allelic richness [20]. The results showed that the most interesting areas in terms of conservation priority are located in Italy, Georgia, and eastern Turkey, thus pointing out the crucial role played by the Italian peninsula in global biodiversity conservation strategies. Different approaches are under development for the conservation of *Castanea* spp., and on farm and ex situ conservation combined with other initiatives represent the first step to prevent the loss of biodiversity [3,4].

Moreover, an increased economic interest for sweet chestnut in the food industry increased the demand for selected varieties, which is also driven by several research studies on the potential positive health benefits that can be derived from the consumption of fresh and processed chestnut products [21,22]. In addition, the recognition of quality certification, such as EU Protected Designation of Origin (PDO) and Protected Geographical Indication (PGI), represent strategic marketing tools that are widely applied in the Italian chestnut market, with particular reference to the “Marrone-type” (MT) cultivars, which stand among the most appreciated varieties by the processing industry worldwide. Consequently, the increased market demand and consumers awareness impose the development of reliable methods for the description and identification of the cultivars [23], and for the characterisation of the fruit traits for the selection of high-quality products (good sensorial and qualitative properties, high bioactive compound content) [24]. For these reasons, several techniques are applied for the characterisation of chestnut cultivars, from the implementation of molecular typing such as SSR markers [25], to morpho-biological, sensory, phenological and chemical analysis. Furthermore, chemical and sensory analyses are on the rise because they allow us to define the most proper technological uses for each cultivar by the study of the nut properties and merceological traits [26]. Moreover, characterisation and conservation of chestnut germplasm represent crucial aspects to ensure adaptability and productivity of the crop, also in relation to the recent climate changes [27].

In this study, a combination of different approaches was applied to describe and characterise 18 chestnut cultivars in order to assess their diversity in bioactive compounds and sensory attributes. It is worth noting that the attempt of silencing the exogenous sources of variability on the phenotypical expression maximises the likelihood of detecting the genotype influence on chestnut chemical and sensorial properties, hence providing an element of novelty and added value to the current state-of-the-art method in the subject of chestnut cultivars characterisation. The diversity that was detected in bioactive compound profiles and sensory traits among the chestnut cultivars is likely related to the effect of the genotype. Thus, chemical and sensorial analyses such as the ones that were applied in this study could be, in the future, an important mean of characterisation, while also allowing the evaluation and the selection of new cultivars [26].

## 2. Materials and Methods

### 2.1. Plant Material and Sampling Site

Samples of *Castanea* spp. fruits were collected in October 2018 from 18 accessions grown in the collection field (about 5 ha) of the Chestnut R&D Center—Piemonte in Chiusa Pesio, Cuneo Province (North-Western Italy) (lat. 44°18′27.5′′; long. 7°40′57.3′′; elevation 575 m above sea level). These cultivars were grown under the same pedoclimatic conditions, agronomic management practices and, for the first time, on the same clonal rootstock. The origin and identification codes of the sampled material are listed in Table 1. 

**Figure 1:**
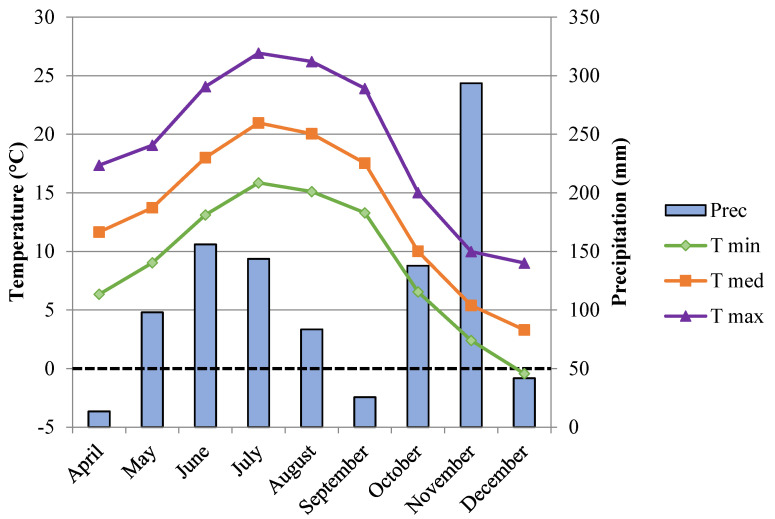
Monthly (maximum, mean, and minimum) temperature and rainfall values available from April to December 2018.

The experimental design allowed the assessment of the genotype effect on the chestnut composition while controlling for the potential confounding effect potentially exerted by the above environmental and agronomic variables. This approach resulted in the implementation of an effective tool to distinguish the different cultivars and, in particular, the “Marrone-type” (MT) cultivars from the other categories as sweet chestnut (SC) and Euro-Japanese hybrids (EH). The germplasm repository includes the main local and Italian chestnut cultivars, several European varieties, and accessions from China, Japan, and USA [27]. The area is characterised by a temperate climate and is located in the phytoclimatic transition zone between “cold *Castanetum*” and “hot *Fagetum*”, following Mayr-Pavari’s classification [4]. The annual mean temperature and precipitations are 13.3 °C and 993 mm, respectively. All climatic data were extracted from the meteorological station placed in the collection field. Figure 1 shows climate data from April to December, the period from vegetative awakening to winter dormancy. Soils are composed of fluvial deposits, with a high concentration of sand, and the soil depth is limited (between 30 and 60 cm) by the presence of coarse gravel [4].

### 2.2. Sample Preparation

Chestnut fruits (1 kg for each plant of each cultivar—three plants for each cultivar) from 18 accessions were randomly collected, hand selected to remove damaged fruits, and divided into two lots for sensory and chemical analysis. The nuts for the sensory analysis were boiled (100 °C for 45 min) and given to the trained panellists for the tasting sessions (see Sensory analysis section).

Raw nuts used for the chemical analysis were hand-pealed, fragmented in small pieces, dried in an oven (WIPA, Stadtlohn, Germany) at 40 °C for 2 days, and then ground to a fine powder and subdivided into small portions (500 g for each one) sealed in plastic bags.

### 2.3. Sensory Analysis

Sensory evaluation was used to identify and quantify the product organoleptic traits [28]. Currently, the technique is widely applied to a large range of food [29], and it is very important for the selection of cultivars to be used by agri-food industry.

Sensory analysis was performed in a specific sensory laboratory, by 12 selected panellists (gender ratio: 50:50; age range: 20–50-year-old) from ONAFrut (National Organization of Fruit Tasters). Three training sessions were carried out with 12 panellists to ensure a common lexicon of terms for flavour and aroma. During the three training sessions, the panellists worked in a group, but they individually evaluated the samples. After each panel training session, a discussion was held to decide the appropriate set of descriptors to use [30]. Quantitative Descriptive Analysis (QDA) was carried out as analytical-descriptive method [31]. Selected descriptors were: ease of peeling, seed colour, intensity of flavour, intensity of sweetness, intensity of saltiness, intensity of bitterness, flouryness, and chestnut aroma. Descriptive terms, definitions, and associated reference standards used in the sensory analysis of chestnuts were reported in Supplementary Table S1. Each descriptor was evaluated on a continuous scale partially structured into 10 segments as reported in literature [31,32,33,34]. Finally, the same scale was used to evaluate the descriptor of personal judgement of each panellist, based on a subjective approval rating.

### 2.4. Extraction Protocols

All the chemicals/reagents are reported in Supplementary Materials. Polyphenolic compounds were extracted with a mixture of methanol: water: 37% HCl (95:4.5:0.5, *v*/*v*/*v*). Methanolic extracts were filtered through a membrane microfilter (polytetrafluoroethylene membrane, PTFE; pore size 0.45 μm), and then were stored for a few days at normal atmosphere (NA), at 4 °C and 95% RH.

Monoterpenes, sugars, and organic acids were extracted with 95% ethanol solution. Samples were then stored until analysis in NA, at 4 °C and 95% RH.

Ascorbic acid and dehydroascorbic acid were extracted by an extraction solution (0.1 mol·L^−1^ citric acid, 2 mmol·L^−1^ ethylenediaminetetraacetic acid (EDTA) disodium salt, and 4 mmol·L^−1^ sodium fluoride in methanol-water, 5:95, *v*/*v*). *o*-Phenylenediamine (OPDA) solution (18.8 mmol·L^−1^) was added to 750 μL of extracted samples for dehydroascorbic acid (DHAA) derivatisation to a fluorophore, 3-(1,2-dihydroxyethyl)furo(3,4-b) quinoxaline-1-one (DFQ).

### 2.5. Spectrophotometric Analysis

Antioxidant capacity in the chestnut fruits was assessed by a ferric reducing antioxidant power (FRAP) assay [35], and results were expressed as millimoles of Fe^2+^ equivalents per kilogram (solid food) of dried weight (DW).

The total polyphenol content (TPC) was evaluated following the Folin–Ciocalteu colourimetric method [36], and the results were expressed as grams of gallic acid equivalents (GAE) per kilogram of DW.

Absorbance at 595 nm (for antioxidant capacity) and 765 nm (for TPC) with a UV/Vis spectrophotometer (1600-PC, VWR International) was recorded.

### 2.6. Chromatographic Analysis

An Agilent 1200 High Performance Liquid Chromatograph, equipped with a G1311A quaternary pump, a manual injection valve, and a 20 μL sample loop, coupled to an Agilent GI315D UV-Vis diode array detector (Agilent Technologies, Santa Clara, CA, USA), was used for the analysis. 

Six different chromatographic methods were used to analyse the samples. Chromatographic separations were carried out on a Kinetex—C18 column (4.6 × 150 mm, 5 μm, Phenomenex, Torrance, CA, USA), and a SphereClone—NH_2_ column (4.6 × 250 mm, 5 μm, Phenomenex, Torrance, CA, USA). Different chromatographic conditions were used to analyse the samples according to the methods described by other studies [37,38], with some modifications, and previously were validated for fresh and dried fruits, herbal medicines, and other food products. Identification and detection were performed with an UV—Vis Diode Array Detector by scanning from 190 to 600 nm. The chromatographic conditions of each method are reported in Table 2. The external standard method was used for quantitative determinations. All results were expressed as g·kg^−1^ of DW.

### 2.7. Data Analysis

Sensory and nutraceutical data of 18 chestnut cultivars were subjected to one-way analysis of variance (ANOVA), and the averages were compared with the Tukey’s HSD post-hoc comparison test (*n* = 3) [39]. Correlation between sensory and phytochemical data was evaluated with Pearson’s coefficient (r) [39]. A principal component analysis (PCA) [39,40] was carried out on the data matrix including 54 rows (3 repetitions for 18 samples) and 11 fields, each one representing a variable obtained from the chemical analyses. Such variables included the content of nine chemical classes CA (cinnamic acids), FL (flavonols), BE (benzoic acids), CAT (catechins), TA (tannins), MO (monoterpenes), OA (organic acids), VC (vitamin C), SU (sugars), the TPC (total polyphenol content), and AA (antioxidant activity). The Bartlett’s test of sphericity was carried out and the Kaiser–Meyer–Olkin (KMO) index was calculated from the data matrix [39,40]. The data matrix was subsequently centred and scaled columnwise and the corresponding cell values were, thus, transformed into Z-scores [41]. Based on the outcomes of the Bartlett’s test of sphericity and of the KMO index, a principal component analysis (PCA) was performed on the transformed data matrix. Varimax rotation of the principal axes was applied [39,40]. The minimum number of principal components (PCs) accounting for at least the 50% of the total variance was retained. The association between the chemical variables and the retained PCs was assessed from the plots displaying the loadings of each chemical variable in the PCs plane [39,40]. Points coordinates in the PCs plane were analysed as reported in Lione et al. (2015) [42] and Lione and Gonthier (2016) [43]. The spatial distribution pattern of all the points plotted in the PCs plane was analysed with the Clark-Evans test [44]. The spatial distribution pattern of the points associated with the MT group in the PC plane was analysed with the Mean Distance Randomisation Test Left Tailed (MDRT_LT_), performed on a subset of 106 permutations [43]. Similarly, the spatial distribution of cases associated with SC and EH groups was assessed by the Mean Distance Randomisation Test Right Tailed (MDRT_RT_), carried out by setting the same permutation number. 

The effect of the genotype on the chemical fingerprint was tested by fitting a conditional inference tree model [45,46] on CA, FL, BE, CAT, TA, MO, OA, VC, SU, TPC, and AA as response variables and on the genotype as predictor. The unbiased recursive partitioning algorithm described in Hothorn et al. (2006) [45] and Hothorn and Zeileis (2015) [46] was used for model fitting. The algorithm was run by setting the Bonferroni *p*-value correction for multiple comparisons and the 95% criterion to implement the model splits [39,45,46].

ANOVA and PCA were performed with statistical software package IBM SPSS Statistics 22.0 (IBM, Armonk, NY, USA), the Mean Distance Randomisation Tests were run with the MDT software (https://apsjournals.apsnet.org/doi/suppl/10.1094/PHYTO-05-15-0112-R) [43], and the conditional inference tree model fitting was performed in R [47] with the package partykit [46]. For all statistical tests, the significance threshold was set at 5%.

## 3. Results and Discussion

### 3.1. Sensory Analysis

Eight sensory attributes were used to characterise, qualitatively and quantitatively, the 18 chestnut cultivars analysed in this study, including three textural and visual (ease of peeling, seed colour, and flouryness) and five flavour descriptors (intensity of flavour, intensity of sweetness, intensity of saltiness, intensity of bitterness, and chestnut aroma). An overall subjective estimation (subjective judgement) was also expressed in order to describe the panellist personal rating. A 0 to 10 linear scale was used to evaluate the intensity of each attribute.

Although different cultivars showed the same sensory attributes, they differed in terms of intensity [48]. Within the SC and MT groups, cultivars of *C. sativa* showed significant differences (*p* < 0.05) for all the descriptors, except for intensity of saltiness. The EH group was more homogeneous and samples differed (*p* < 0.05) only for ease of peeling, seed colour, intensity of flavour, and intensity of bitterness.

**Figure 2:**
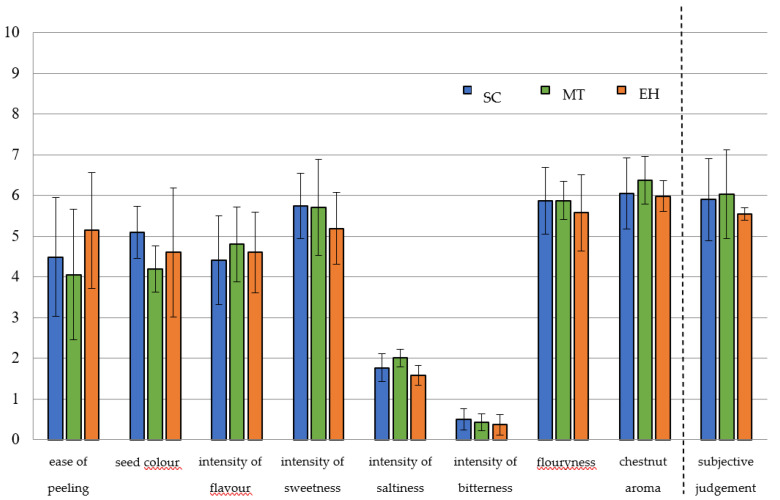
Sensory profiles for analysed chestnut groups (Sweet chestnut—SC, Marrone-type—MT, Euro-Japanese hybrid—EH). *Y*-axis represents the intensity value of sensory descriptors in a continuous scale partially structured into 10 segments.

SC cultivars achieved high values in terms of seed colour and intensity of sweetness, according to Kunsch et al. [49], but they were the bitterest ones, with low values for intensity of flavour, a descriptor very appreciated by consumers (Figure 2). 

Significant differences (*p* < 0.05) were observed between chestnut samples for ease of peeling and seed colour, two important attributes. ‘Marrubia’ (SC) presented the highest value of ease of peeling (7.00 ± 0.65), while ‘Marrone di Castel del Rio’ and ‘Marrone di Marradi IGP’ showed a good ease of peeling in the MT group (5.21 ± 2.32 and 5.67 ± 1.94, respectively) as shown in other studies [48]. ‘Marsol’ presented the lowest ease of peeling value (3.50 ± 0.71) for the EH group, in particular if compared to ‘Bouche de Betizac’ (5.86 ± 2.14), which was in agreement with the values reported in other studies [49]. A sensory profile of all the chestnut samples is reported in Table 3.

**Figure 3:**
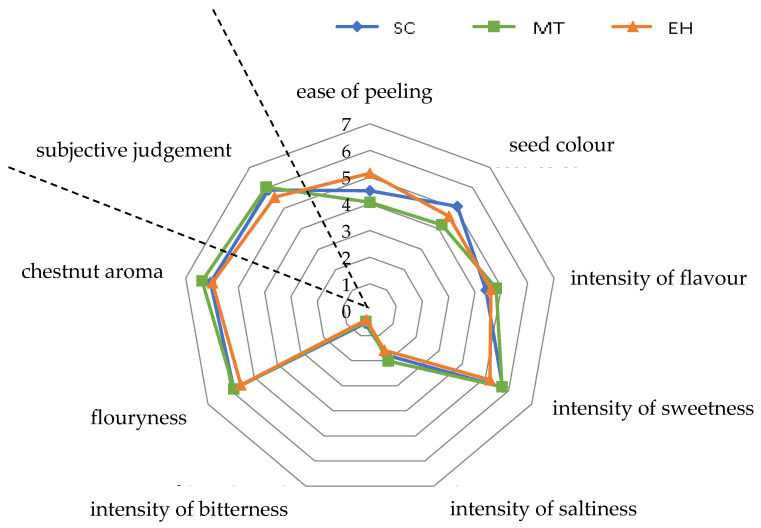
Radar chart for sensory analysis of considered chestnut groups (Sweet chestnut—SC, Marrone-type chestnut—MT, Euro-Japanese hybrid—EH). Subjective judgement is not part of radar chart for sensory analysis described by Quantitative Descriptive Analysis (QDA), but it was added together to other descriptors as complementary information.

MT cultivars are commonly appreciated for fresh and processing consumption thanks to their positive traits (kernel easily separable from episperm, ease of seed peeling, reddish colour epicarp, good sweet flavour). Sensory analysis on the three cultivars from this group partially confirmed the results published in previous studies [48,50]. They showed the highest ratings for intensity of saltiness and chestnut aroma. Intensity of sweetness level was also considerably high, and it was comparable with the other SC chestnuts (Figure 3). 

In particular, the descriptor “intensity of sweetness” varied significantly (*p* < 0.05) among the SC and MT cultivars: ‘Canepina’ presented the lowest value (4.36 ± 1.18) and ‘Marrone di Castel del Rio’ the highest one (7.07 ± 1.59), while there were no significant differences (*p* > 0.05) among the EH group. Data showed a high correlation level, evaluated by Pearson’s coefficient (r), between intensity of sweetness and sugar content in the analysed cultivars (*r* = 0.71 for SC, *r* = 0.89 for MT, and *r* = 0.81 for EH). ‘Marrubia’ showed no intensity of bitterness, recording the lowest value for this attribute, while ‘Marsol’ was the most bitter Euro-Japanese hybrid (0.90 ± 0.74). A high correlation coefficient was also found between intensity of bitterness and tannin content (*r* = 0.73 for SC, *r* = 0.68 for MT, and *r* = 0.85 for EH).

Significant differences (*p* < 0.05) were also observed in intensity of flavour and chestnut aroma (flavour descriptors). The first one is usually measured during the seed breakup and refers to the smell sense, while the other is the expression of chestnut aroma measurable by multiple senses as described in other studies [48,51]. Results highlighted a high level of intensity of flavour in the ‘Marrubia’ cultivar (6.21 ± 1.38), significantly higher (*p* < 0.05) than all the other sweet chestnuts. In the case of chestnut aroma, both ‘Marrubia’ and ‘Marrone di Castel del Rio’ significantly differed (*p* < 0.05) from the other cultivars that showed higher ratings. Euro-Japanese hybrids did not differ significantly (*p* > 0.05) in terms of chestnut aroma, while significant differences (*p* < 0.05) were observed in intensity of flavour, which was higher in ‘Marsol’ (6.00 ± 0.23).

Panellists were also asked to give a personal preference to each cultivar or hybrid, although not planned in the Quantitative Descriptive Analysis (QDA), in order to assess a subjective general rating. ‘Marrubia’ (7.71 ± 0.76) and ‘Contessa’ (7.10 ± 1.30) for the SC chestnuts, and ‘Marrone di Castel del Rio’ (7.43 ± 1.24) for the MT group, displayed significantly higher (*p* < 0.05) values than all the other cultivars, while EH chestnuts showed lower but not significant values (*p* > 0.05) in the group. Data pointed out a good correlation between judgment and sugar content (*r* = 0.57 for SC, *r* = 0.89 for MT, and *r* = 0.73 for EH). These values seemed to be lower than the Pearson’s coefficients between intensity of sweetness and sugar content due to the influence of other sensory traits on panellist judgment. Indeed, a significant correlation was also observed between judgment and chestnut aroma, with values similar to the correlation between judgment and sugar content (*r* = 0.62 for SC, *r* = 0.80 for MT, and *r* = 0.78 for EH). These results showed that judgment was equally influenced by chestnut aroma and sugar content. Moreover, a significant correlation between intensity of sweetness and chestnut aroma was observed (*r* = 0.52 for SC, *r* = 0.72 for MT, and *r* = 0.54 for EH). In any case, further statistical assessments are necessary to confirm this hypothesis. Moreover, a good correlation was found between intensity of sweetness and judgement (*r* = 0.56 for SC, *r* = 0.873 for MT, and *r* = 0.89 for EH group), suggesting the influence of this sensory parameter on panellist personal preference. Pearson’s correlation data were reported in Supplementary Table S2. Even if MT cultivars were very floury and with dark seed colour, they were the most appreciated chestnuts according to the personal panellist judgment, confirming the findings of Mellano et al., 2007 [52]. Euro-Japanese hybrids, widely spread and cultivated because of their resistance to diseases and their high kernel quality [53], scored as the easiest to peel and the lowest in terms of flouryness, intensity of bitterness, and intensity of saltiness. Nevertheless, hybrids showed values lower than other cultivars for intensity of sweetness and chestnut aroma, as remarked by the low score assigned by the panellists. 

### 3.2. Phytochemical Composition, Antioxidant Capacity, and Nutritional Properties

The phytochemical and nutritional profile (contents of polyphenols, monoterpenes, vitamin C, organic acids, and sugars), complemented by the measurement of the antioxidant capacity, of the 18 cultivars and hybrids of chestnuts were defined by chemical analysis [54].

**Figure 4:**
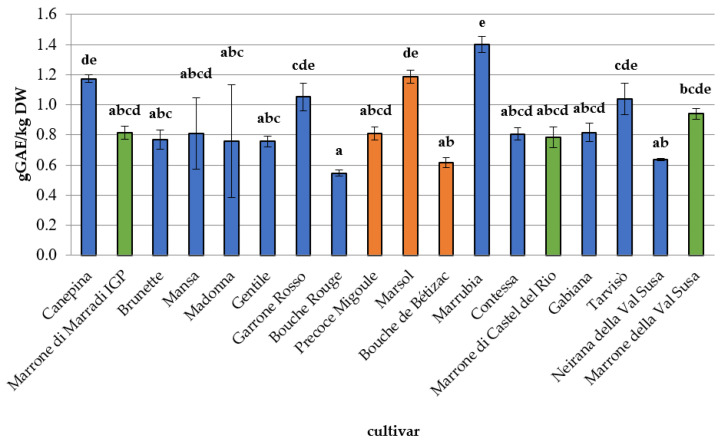
Total polyphenol content of the 18 chestnut cultivars. Different letters for each cultivar indicate the significant differences at *p* < 0.05. Blue colour: Sweet chestnut—SC; orange colour: Euro-Japanese hybrid—EH; green colour: Marrone-type chestnut—MT.

**Figure 5:**
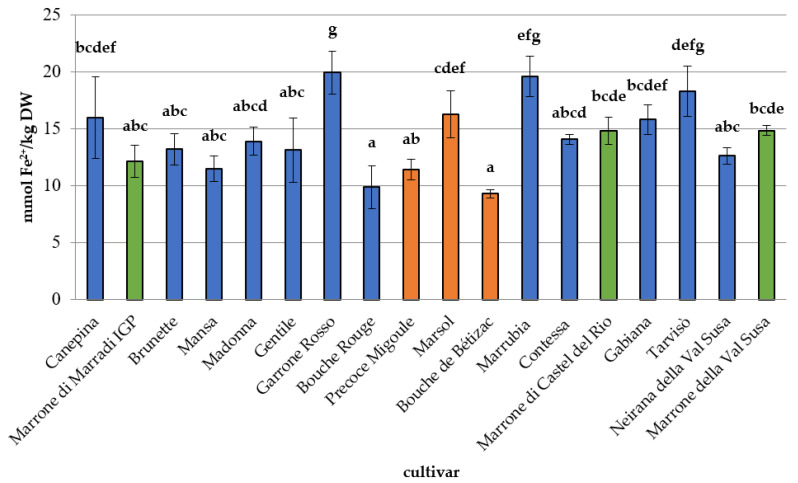
Antioxidant activity of the 18 chestnut cultivars. Different letters for each cultivar indicate the significant differences at *p* < 0.05. Blue colour: Sweet chestnut—SC; orange colour: Euro-Japanese hybrid—EH; green colour: Marrone-type chestnut—MT.

Mean TPC and antioxidant capacity values are reported in Figure 4 and Figure 5. TPC values (Figure 4) ranged from 0.55 ± 0.02 g_GAE_ kg^−1^ DW for the French cultivar ‘Bouche Rouge’ to 1.40 ± 0.05 g_GAE_ kg^−1^ DW for the Italian cultivar ‘Marrubia,’ in agreement with other studies [55,56]. Chestnut TPC values were similar or higher than values detected in other tree nuts [57]. The highest phenolic content was observed for the Piemonte Region cultivars. Significantly different antioxidant capacity values (*p* < 0.05), expressed as a FRAP assay, were observed among the cultivars with a trend similar to the one observed for TPC levels. Antioxidant capacity ranged from 9.30 ± 0.39 mmol Fe^+2^ kg^−1^ DW (‘Bouche de Bètizac’) to 19.96 ± 1.89 mmol Fe^+2^ kg^−1^ DW (‘Garrone Rosso’), as shown in Figure 5, in agreement with previous studies [51,58]. Chestnuts are a potential source of bioactive molecules, with a good antioxidant capacity, as highlighted by similar studies [51,59]. However, establishing the contribution of each single bioactive compound to the total antioxidant activity may be difficult because of the synergistic combination and interaction between the different substances (phytocomplex). Each antioxidant compound may improve the effectiveness of the others, and this action could influence the overall response (total antioxidant capacity) [60]. This additive effect may explain the significant differences between the antioxidant activities of the different analysed samples; for this reason, samples with the highest values of TPC and vitamin C did not always show the highest antioxidant capacity.

**Figure 6:**
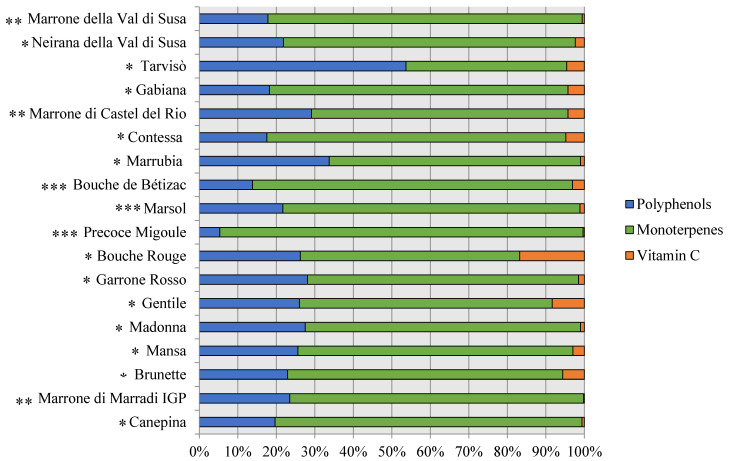
Phytocomplex representation of the analysed chestnut cultivars. The colours identify the different classes: blue corresponds to polyphenols, green to monoterpenes, and orange to vitamin C. * Sweet chestnut (SC). ** Marrone-type chestnut (MT). *** Euro-Japanese hybrid (EH).

The phytochemical composition of the analysed cultivars identified 21 biomarkers by HPLC-DAD. To evaluate the contribution of each class to the total phytocomplex composition, the bioactive compounds were grouped in the following classes: polyphenols (as the sum of cinnamic acids, flavonols, benzoic acids, catechins, and tannins), monoterpenes, and vitamin C (mean values were considered) (Figure 6).

Monoterpenes, recognised for their anti-tumour and anti-inflammatory properties [61], represented the main component of the phytocomplex, reaching the 88% of EH cultivars, followed by polyphenols, characterised by antioxidant, anti-bacterial, and anti-tumour properties [62], and is between 10 and 25% for EH and SC, respectively, and has vitamin C in low quantities (2%). However, the cultivar ‘Tarvisò’ showed a higher proportion of polyphenols than monoterpenes. The highest levels of polyphenols and monoterpenes were detected in ‘Canepina’ (0.19 g·kg^−1^ DW and 0.76 g·kg^−1^ DW, respectively), a cultivar from central Italy, while the highest values of vitamin C were observed in the French cultivar ‘Bouche Rouge’ (0.19 g·kg^−1^ DW) as shown in Table 4. 

**Figure 7:**
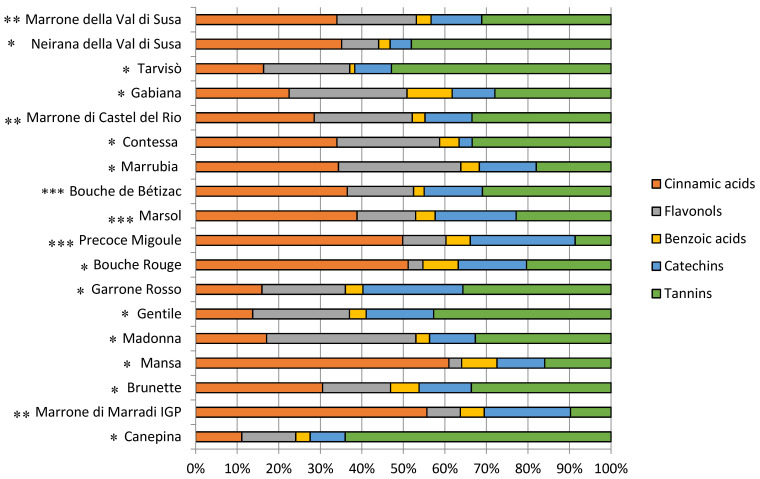
Polyphenol profile of the analysed chestnut cultivars. * Sweet chestnut (SC). ** Marrone-type chestnut (MT). *** Euro-Japanese hybrid (EH).

Within the polyphenolic group, differences were observed among chestnut genotypes, but most of the cultivars showed phenolic levels similar to hazelnut ones [63,64], and higher than walnut ones [65]. Cinnamic acids and flavonols were the most important classes among phenolics (20–40% and 15–20%, respectively), followed by tannins (27%, 22%, and 43% for MT, EH, and SC chestnuts, respectively) as shown in Figure 7. Flavonols, catechins, and benzoic acids were detected in very low quantities (<10%).

Data on each bioactive and nutritional compound content are reported in Supplementary Table S3. Tannins were the main polyphenolic class in the analysed chestnuts, followed by cinnamic acids and flavonols, the latter with a great variability in the results. The ‘Canepina’ cultivar displayed high tannin levels (1.20 ± 0.35 g·kg^−1^ DW). This value was higher than the average of the other samples, as confirmed by Tukey’s test (*p* < 0.05), which included this sample in a separate group. The same holds true for the catechin class, which was represented mainly by epicatechin (0.15 ± 0.04 g·kg^−1^ DW). Regarding catechins, the MT varieties were included within the same group, together with ‘Gentile’ and ‘Madonna’ cultivars, that showed relevant quantities of these compounds as shown by De Biaggi et al. (2018) [51]. The identification of catechin and epicatechin is an important result as they are involved in the inhibition of lipid peroxidation, and inhibition of human cancer cell line proliferation and cyclooxygenase enzymes [66]. For tannins, except for ‘Canepina’ and ‘Tarvisò’ (the highest values) and ‘Mansa’ and ‘Marrone di Marradi IGP’ (the lowest values), all the samples contained between 0.10 and 0.25 g·kg^−1^ DW, while French cultivar ‘Bouche Rouge’, with about 0.06 g·kg^−1^ DW, was classified by the post-hoc test in a separate group (*p* < 0.05). The presence of tannins in adequate amounts increases the nutraceutical properties of chestnuts since these compounds are free radical quenchers [67].

Caffeic and coumaric acids were quantified in all the samples, although in low quantities (<0.01 g·kg^−1^ DW). Ferulic acid was not detected in all the analysed chestnut cultivars, except in ‘Canepina’ and ‘Madonna’ (about 0.01 g·kg^−1^ DW). In the flavonol class, no traces of quercetin were detected in any of the analysed samples, and quercitrin and rutin occurred only at low concentrations (always below 0.01 g·kg^−1^ DW), except in ‘Marrubia’, which showed a quercitrin value of 0.120 ± 0.001 g·kg^−1^ DW. The most representative flavonol was isoquercetin, which is detected in large quantities, especially in ‘Tarvisò’ (0.26 ± 0.03 g·kg^−1^ DW), ‘Canepina’ (0.23 ± 0.03 g·kg^−1^ DW), and ‘Madonna’ (0.22 ± 0.04 g·kg^−1^ DW). ‘Tarvisò’ cultivar displayed the highest flavonol content (0.26 ± 0.03 g·kg^−1^ DW), followed by ‘Madonna’ (0.25 ± 0.04 g·kg^−1^ DW), and ‘Canepina’ (0.24 ± 0.03 g·kg^−1^ DW), while ‘Mansa’, ‘Bouche Rouge’, and ‘Marrone di Marradi IGP’ showed lower values. Flavonols quench active oxygen species and inhibit in vitro oxidation of low-density lipoproteins [68].

Ellagic acid was the most abundant benzoic acid in ‘Gabiana’ and ‘Canepina’ (about 0.05 g·kg^−1^ DW), while gallic acid showed high variability among the samples, except for ‘Marrone della Val Pellice,’ which did not contain gallic acid and, in general, was characterised by the lowest content of benzoic acids. These molecules are very important in human nutrition and are related to many biological properties, including anticancer, anti-atherosclerotic, anti-inflammatory, antihepatotoxic, and anti-HIV replication activities [69].

As well as polyphenols, the analysed samples also showed different monoterpenes and discrete contents of vitamin C, as reported in Supplementary Table S3. Monoterpenes are a large class of naturally bioactive molecules used extensively for their aromatic qualities combined with their antioxidant capacity and anti-inflammatory properties [70]. Many of these molecules have antibacterial and antitumor activity [71]. EH cultivars showed high contents of monoterpenes, in particular ‘Precoce Migoule,’ but ‘Canepina’ (SC) was characterised by the highest content of these compounds (0.76 ± 0.18 g·kg^−1^ DW). Limonene was predominant and reached quantities of 6.35 ± 1.77 g·kg^−1^ DW in the ‘Canepina’ cultivar. High limonene amounts were also found in the EH group. γ-terpinene was detected in high quantities, in particular in ‘Precoce Migoule’ (0.38 ± 0.70 g·kg^−1^ DW), ‘Marrone della Val Pellice’ (0.21 ± 0.13 g·kg^−1^ DW), and ‘Gabiana’ (0.16 ± 0.05 g·kg^−1^ DW), similar to other studies [62,72]. Several studies reported their chemopreventive activity against rodent mammary, skin, liver, lung, and forestomach cancers [73]. Terpinolene, sabinene, and phellandrene were also identified even if at trace levels. 

Vitamin C was evaluated as the sum of ascorbic and dehydroascorbic acids due to their biological activity in human organisms as reported in other studies [60,74]. The maximum vitamin C value was detected in the ‘Bouche Rouge’ cultivar (0.19 ± 0.03 g·kg^−1^ DW), followed by ‘Marrone della Val Pellice’ (0.18 ± 0.09 g·kg^−1^ DW), while the minimum amount was detected in ‘Marrone di Marradi IGP’. Among EH chestnuts, ‘Bouche de Bétizac’ provided the largest amount of vitamin C (0.12 ± 0.02 g·kg^−1^ DW), which was comparable to the values reported in De Biaggi et al. (2018) [51]. The majority of the chestnut cultivars showed vitamin C content similar to walnut and almond ones [57,75], and higher than hazelnut varieties [76].

Large and significant differences (*p* < 0.05) in organic acid and sugar content values were detected among cultivars. Mean values for SC, MT, and EH chestnuts are reported in Table 5. 

High levels of organic acids were observed in hybrids, in particular for ‘Precoce Migoule’ (7.43 ± 0.09 g·kg^−1^ DW), while the chestnut cultivar ‘Garrone Rosso’ showed the lowest values (1.20 ± 0.21 g·kg^−1^ DW). Citric acid was the most abundant organic acid in the analysed chestnuts, with high levels in ‘Mansa’ and ‘Precoce Migoule’ cultivars (5.31 ± 0.25 g·kg^−1^ DW and 3.28 ± 0.25 g·kg^−1^ DW, respectively), followed by quinic acid (maximum value of 3.55 ± 0.28 g·kg^−1^ DW in ‘Brunette cultivar’), as reported by similar studies [77]. Quinic acid was detected in all the samples, except in ‘Marrubia,’ which only contained citric acid. Malic acid was not detected in the analysed chestnuts, due to the intrinsic characteristics of the considered varieties and the effect of the drying treatment applied during the sample preparation [49]. Tukey’s test highlighted significant differences (*p* < 0.05) in the organic acid composition among different cultivars of the SC, MT, and EH groups, leading to the identification of different groups composed by one or a few compounds. This result could be due to the differences associated with the different genotypes but, since organic acids are volatile molecules, other factors could have slightly influenced the results, such as the extraction technique, sample storage, and applied drying technique [78].

The highest quantity of sugars was observed for the cultivar ‘Mansa’ (273.38 ± 21.16 g·kg^−1^ DW), while the average values ranged from 10.64 ± 2.33 g·kg^−1^ DW (‘Bouche Rouge’) to 114.80 ± 10.63 g·kg^−1^ DW (‘Canepina’); chestnut cultivars showed higher values if compared to the sugar levels of other tree nuts such as walnut and almond [79]. MT chestnuts showed sugar levels (15–30 g·kg^−1^ DW) similar to other chestnut cultivars, which was in agreement with other studies [51,80,81]. Although sucrose was the most abundant sugar in many analysed chestnuts, some samples, including ‘Marrone di Marradi IGP,’ ‘Marsol,’ ‘Precoce Migoule,’ ‘Brunette,’ ‘Garrone Rosso,’ ‘Marrone della Val Pellice,’ and ‘Marrubia,’ showed higher contents of glucose than sucrose. Other cultivars (e.g., ‘Mansa,’ ‘Marrone della Val di Susa,’ and ‘Neirana della Val di Susa’) showed higher contents of fructose than glucose. The higher fructose amount compared to the glucose one may be important to define chestnuts as a potential functional food for consumers suffering from type 2 diabetes, as fructose shows a lower glycemic index than glucose and, consequently, the postprandial glycemic peak due to fructose is lower than the glycemic peak due to glucose, as well as the insulin response [82]. As evidenced by the Tukey’s test, the ‘Mansa’ cultivar significantly differed (*p* < 0.05) from the other samples, followed by ‘Canepina’, as mentioned above, and ‘Brunette’ and ‘Gentile’, which reported quantities close to 50 g·kg^−1^ DW. 

### 3.3. Multivariate Analysis

**Figure 8:**
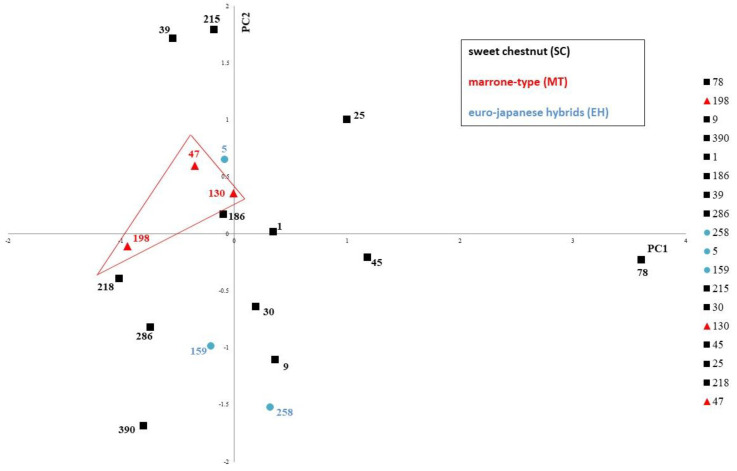
Principal component analysis (PCA) score plot of analysed chestnut cultivars. Mean values (*n* = 3) were considered for each cultivar. Cultivar name (ID): Bouche de Bétizac (159); Bouche Rouge (286); Brunette (9); Canepina (78); Contessa (30); Gabiana (45); Garrone Rosso (39); Gentile (186); Madonna (1); Mansa (390); Marrone di Castel del Rio (130); Marrone di Marradi IGP (198); Marrone della Val di Susa (47); Marrubia (215); Marsol (5); Neirana della Val di Susa (218); Precoce Migoule (258); Tarvisò (25).

Rather than hinging on the action of a single compound, therapeutic effects obtainable from the consumption of fresh fruit and derived-products are the result of the synergistic or additive interaction of several phytochemicals that jointly contribute to disease prevention [83]. For this reason, compounds belonging to the same chemical class were combined in bioactive classes for multivariate data analysis. The outcome of the Bartlett’s test of sphericity (*p* < 0.05) showed a significant collinearity among variables. The KMO index attained a value of 0.74. The PCA resulted in two principal components accounting for 50.71% of the total variance, with 32.47% explained by PC1, and 18.24% by PC2. The location in the PCs plane of the 18 samples (mean values of three repetitions for each cultivar) in relation to phytochemical composition, nutritional properties, and nutraceutical traits is shown in the score plot (Figure 8). 

**Figure 9:**
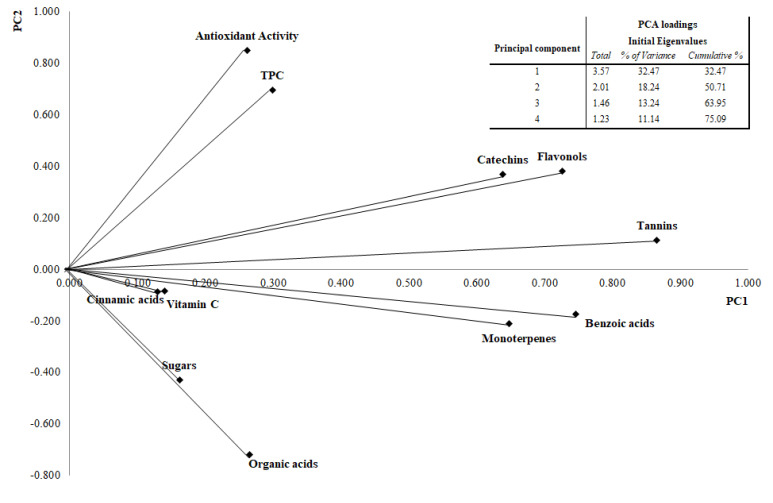
PCA loading plot of considered variables.

PCA showed that cultivars belonging to the MT group, which are highlighted in Figure 8, presented similar traits according to the chemical results. PCA loadings plot showed an association between polyphenolic classes, vitamin C, monoterpenes and PC1, and a correlation between TPC, antioxidant activity, organic acids, sugars, and PC2 (Figure 9). 

Phenolic acids and tannins, associated with PC1, were identified as bioactive classes with the most discriminating power among different genotypes; these phytochemical classes included compounds displaying significant differences (*p* < 0.05) in their bioactive content among the different cultivars. Moreover, monoterpenes showed also a good discriminating power among chestnut samples. For this reason, all these molecules could represent the most important markers in order to build a discriminant model between chestnut genotypes, but further studies are necessary to confirm this hypothesis.

In this study, a multivariate analysis as PCA allowed for the visualisation of the information included in the fingerprints. The results showed that PCA classification characterised the samples according to the different chemical composition, providing information on the bioactive classes and chemical markers that most influence the phytocomplex. A chemometric method was applied with the HPLC fingerprint technique for a better recognition of the analysed extracts as reported by Cirlini et al. [84]. Different marker compounds were detected as the variables most relevant for the discrimination of chestnut cultivars, which could be applied to accurate composition control of a chestnut flour derived from a specific cultivar. In this study, PCA results showed that MT genotypes formed a single group within a larger group of SC and EH cultivars: the HPLC fingerprint combined with chemometrics could be considered as a tool of traceability in order distinguish different genotypes by their phytochemical composition and antioxidant properties, as is reported in other research [85,86].

**Figure 10:**
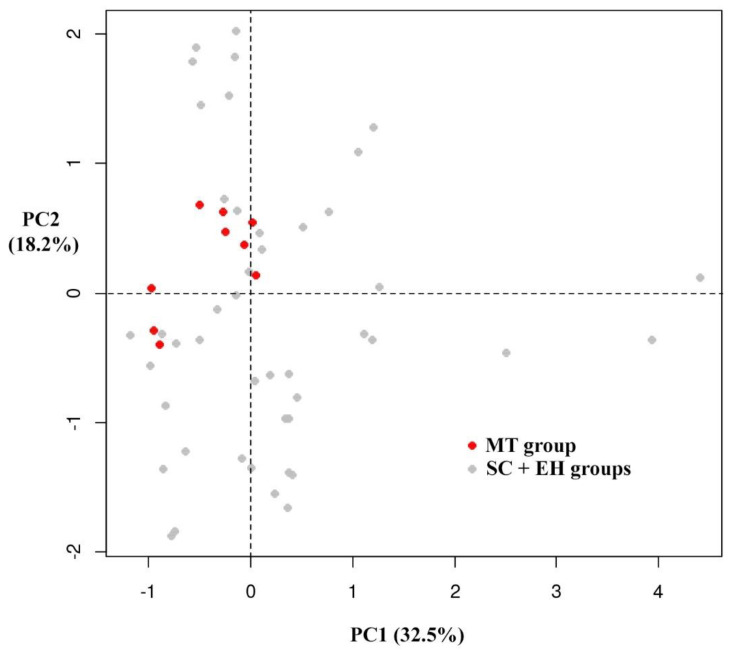
Distribution of the points associated with “Marrone” and “Chestnut” groups in the principal components (PCs) plane. A significant clustering of points associated with MT group (*p* < 0.05) is observed in the PCs plane, while the points associated with SC + EH groups are characterised by a significantly dispersed spatial distribution (*p* < 0.05) in the same PCs plane. Sweet chestnut = SC. Marrone-type chestnut = MT. Euro-Japanese hybrid = EH.

The tests about the spatial distribution pattern further supported the interpretation of the PCA results. The Clark-Evans test showed that the points in the PCs plane were significantly clustered (*r* = 0.643, *p* < 0.05). The MDRT_LT_ test showed a significant clustering of points associated with MT group (*p* < 0.05) in the PC plan, while the MDRT_RT_ test showed that the points associated with SC + EH groups are characterised by a significantly dispersed spatial distribution (*p* < 0.05) in the same PCs plane. These results suggest that the MT cultivars represented a homogenous group with less variable traits than the SC + EH groups (Figure 10). 

In the PCs plane, spatial proximity between points is interpretable in terms of similarity of the underlying features [42]; hence, MT genotypes, closely located in the PCA plane, analysed in this study are characterised by a similar profile of chemical composition and of the associated properties beneficial to human health. The reduced variability in terms of chemical composition of MT cultivars that was pointed out by the geostatistical tests suggests that MT fruits are more suitable for specific uses than SC and EH ones. For instance, quality tracking and certifications are likely easier to be obtained by fruits showing constant chemical and organoleptic features than by fruit whose characteristics may undergo large fluctuations. Moreover, strict dietary requirements might be more likely respected by the consumers if the food they eat is endowed with a stable composition. This holds true not only for direct consumption, but also for the industrial transformation, in general aiming at producing standardised products with constant nutritional and chemical properties. 

The outcomes of the conditional inference tree model pointed out that the genotype plays a significant (*p* < 0.05) role and may account for most of the differences detected among the chemical fingerprints of the chestnut fruits. In fact, the tree model (Supplementary Figure S1) displayed four highly significant (*p* < 0.001) splits resulting in two intermediate and four terminal nodes. 

Each terminal node clustered the genotypes whose overall chemical fingerprint was homogeneous (*p* > 0.05), while genotypes included in separate nodes were characterised by significantly different chemical fingerprints (*p* < 0.05). It is worth noting that while the PCA clearly pointed out the role played by different chemical compounds in profiling, the samples analysed, the conditional inference tree model accounted for the role of the genotype, a categorical variable that cannot be included directly within a PCA, since this can only handle continuous covariates [39,40]. In combination, both analyses strongly support our hypothesis about the genotype influence on the chemical composition of chestnut cultivars grown on the same clonal rootstock and in the same agri-environmental conditions. Although replicates within genotypes were not particularly abundant, the algorithm run to fit the conditional inference tree model [45,46] has been specifically designed to be robust and reliable. Even when the number of covariates is high, the sample size is low or the data are unbalanced, as confirmed by a recent study on chestnut [87].

The combination of chromatographic fingerprint and chemometric evaluation could be a potential tool for chestnut product traceability and quality control, in order to select the best raw material based on the desired traits and properties. In addition, the above tools could be used to avoid potential voluntary or involuntary adulterations and contaminations. These hyphenated techniques could also contribute to the analysis of several processed products. Chemical fingerprint coupled to chemometrics could also be a useful tool to obtain label certifications for the valorisation of specific local genotypes. Moreover, the approach used in this study could also contribute to the selection of new varieties more tolerant or resistant to pests and diseases, in particular considering the new issues related to the climate change, and to support and improve breeding programs and preservation strategies for the existing cultivars.

## 4. Conclusions

Chromatographic and spectrophotometric data confirmed the high variability within the genus *Castanea*, and multivariate analysis allowed to explain such variability in terms of phytochemical and nutritional composition, characterising the different genotypes. Monoterpenes, important for their anti-inflammatory and anti-tumour activities, represented the main component of the chestnut composition (in particular, 88% for EH cultivars); followed by polyphenols, which are characterised by antioxidant and anti-bacterial properties (10–25% for EH and SC); and vitamin C in trace (about 2%). Tannins were the main polyphenolic compounds detected in the analysed chestnuts, followed by phenolic acids and flavonols. In particular, ‘Canepina’ presented higher phenolic amounts than almost all the analysed cultivars. Moreover, the majority of the analysed chestnut cultivars showed a content of bioactive compounds, as phenolics and vitamin C, whose levels were similar to, or higher than those reported for the main hazelnut, walnut, and almond varieties.

As genetic and phytochemical diversity represent fundamental aspects to ensure the productivity and adaptability of chestnut orchards, different approaches need to be developed to ensure the correct characterisation strategy. Hence, different techniques were combined in this study to define a suitable strategy for the characterisation of the chestnut cultivars as a key prerequisite to allow the conservation of *Castanea* germplasm. 

The analysed cultivars were selected as part of a core collection that maximises the chestnut agro-biodiversity. The diversity observed among the analysed cultivars could be strictly associated to the genotype effect and underlines the large variability of the genus *Castanea*, and therefore, the importance of in farm and ex situ conservation of local germplasm as part of a global strategy, and also in relation to an active utilisation of agrobiodiversity.

## Figures and Tables

**Table 1:** Origin and identification codes (number of the tree in the orchard) of the sampled raw material.

Category	Species	Cultivar	Nut	ID	Country
Sweet chestnut (SC)	*C. sativa*	Canepina	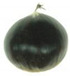	78	Italy
	*C. sativa*	Brunette	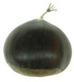	9	Italy
	*C. sativa*	Mansa	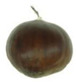	390	Spain
	*C. sativa*	Madonna	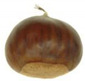	1	Italy
	*C. sativa*	Gentile	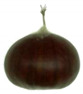	186	Italy
	*C. sativa*	Garrone Rosso	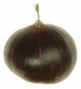	39	Italy
	*C. sativa*	Bouche Rouge	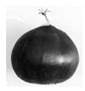	286	France
	*C. sativa*	Marrubia	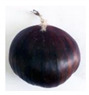	215	Italy
	*C. sativa*	Contessa	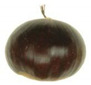	30	Italy
	*C. sativa*	Gabiana	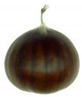	45	Italy
	*C. sativa*	Neirana della Val di Susa	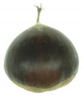	218	Italy
	*C. sativa*	Tarvisò	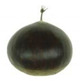	25	Italy
Marrone-type chestnut (MT)	*C. sativa*	Marrone di Castel del Rio	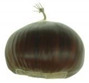	130	Italy
	*C. sativa*	Marrone di Marradi IGP	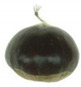	198	Italy
	*C. sativa*	Marrone della Val di Susa	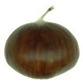	47	Italy
Euro-Japanese hybrid (EH)	*C. sativa* x *C. crenata*	Precoce Migoule	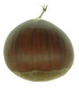	258	France
	*C. sativa* x *C. crenata*	Marsol	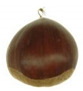	5	France
	*C. sativa* x *C. crenata*	Bouche de Bétizac	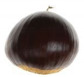	159	France

**Table 2:** Chromatographic conditions of the used methods.

Method	Compounds of Interest	Stationary Phase	Mobile Phase	Flow (mL min^−1^)	Wavelength (nm)
A	cinnamic acids, flavonols	KINETEX—C18 column (4.6 × 150 mm, 5 μm)	A: 10 mM KH_2_PO_4_/H_3_PO_4_, pH = 2.8	1.5	330
			B: CH_3_CN		
B	benzoic acids, catechins,	KINETEX—C18 column (4.6 × 150 mm, 5 μm)	A: H_2_O/CH_3_OH/HCOOH (5:95:0.1 *v*/*v*/*v*), pH = 2.5	0.6	280
	Tannins		B: CH_3_OH/HCOOH (100:0.1 *v*/*v*)		
C	monoterpenes	KINETEX—C18 column (4.6 × 150 mm, 5 μm)	A: H_2_O	1.0	250
			B: CH_3_CN		
D	organic acids	KINETEX—C18 column (4.6 × 150 mm, 5 μm)	A: 10 mM KH_2_PO_4_/H_3_PO_4_, pH = 2.8	0.6	214
			B: CH_3_CN		
E	vitamins	KINETEX—C18 column (4.6 × 150 mm, 5 μm)	A: 5 mM C_16_H_33_N(CH_3_)_3_Br/50 mM KH_2_PO_4_, pH = 2.5	0.9	261, 348
			B: CH_3_OH		
F	sugars	SphereClone—NH_2_ column (4.6 × 250 mm, 5 μm)	A: H_2_O	0.5	286
			B: CH_3_CN		

Method A—gradient analysis: 5% B to 21% B in 17 min + 21% B in 3 min (2 min conditioning time). Method B—gradient analysis: 3% B to 85% B in 22 min + 85% B in 1 min (2 min conditioning time). Method C—gradient analysis: 30% B to 56% B in 15 min + 56% B in 2 min (3 min conditioning). Method D—gradient analysis: 5% B to 14% B in 10 min + 14% B in 3 min (2 min conditioning time). Method E—isocratic analysis: ratio of phase A and B: 95:5 in 10 min (5 min conditioning time). Method F—isocratic analysis: ratio of phase A and B: 5:85 in 12 min (3 min conditioning time).

**Table 3:** Sensory profiles of the analysed chestnuts.

Cultivar	Cv Type	ID	Ease of Peeling	Seed Colour	Intensity of Flavour	Intensity of Sweetness	Intensity of Saltiness	Intensity of Bitterness	Flouryness	Chestnut Aroma	Subjective Judgement
Bouche de Betizac ***	EH	159	5.86 ± 2.14 ab	2.64 ± 1.97 b	3.71 ± 1.60 b	6.07 ± 1.30 a	1.43 ± 0.98 a	0.29 ± 0.49 ab	6.86 ±0.80 a	6.00 ± 1.55 a	5.43 ± 2.24 a
Brunette *	SC	9	4.21 ± 2.12 abcd	5.21 ± 0.99 ab	3.93 ± 2.01 ab	6.14 ± 0.85 abc	1.43 ± 1.27 a	0.21 ± 0.39 ab	5.36 ± 1.93 ab	5.50 ± 1.12 ab	5.79 ± 1.07 abcd
Canepina *	SC	78	3.43 ± 1.30 bcd	4.93 ± 0.84 ab	3.00 ± 1.15 b	4.36 ± 1.18 b	1.07 ± 1.02 a	0.43 ± 0.79 ab	6.71 ± 1.15 a	5.14 ± 0.90 b	4.86 ± 1.35 cd
Contessa *	SC	30	4.65 ± 1.73 abcd	5.90 ± 1.78 ab	5.65 ± 1.93 ab	6.85 ± 1.70 abc	1.97 ± 1.13 a	0.00 ± 0.00 b	6.35 ± 1.06 ab	7.35 ± 1.60 ab	7.10 ± 1.30 abcd
Gabiana *	SC	45	5.33 ± 2.06 abcd	4.44 ± 1.51 ab	3.39 ± 1.32 ab	5.50 ± 1.70 abc	1.78 ± 1.30 a	1.22 ± 0.79 a	3.94 ± 1.24 b	4.72 ± 1.95 b	4.56 ± 2.07 d
Garrone Rosso *	SC	39	5.43 ± 2.15 ab	5.21 ± 1.35 ab	5.21 ± 2.48 ab	5.86 ± 1.70 abc	1.71 ± 0.49 a	0.29 ± 0.76 ab	6.86 ± 1.35 a	6.79 ± 1.47 ab	7.07 ± 1.02 abcd
Gentile *	SC	186	5.11 ± 1.27 abcd	3.56 ± 0.98 b	4.33 ± 1.22 ab	4.72 ± 1.20 bc	1.94 ± 0.73 a	0.78 ± 0.79 ab	6.06 ± 1.67 ab	5.78 ± 1.99 ab	6.00 ±1.58 abcd
Mansa *	SC	390	3.13 ± 1.64 bcd	5.13 ± 1.46 ab	3.13 ± 0.79 b	6.00 ± 1.60 abc	1.94 ± 1.21 a	1.31 ± 1.03 a	6.13 ± 2.10 ab	6.19 ± 1.13 ab	5.17 ± 1.12 abcd
Marrone di Castel del Rio **	MT	130	5.21 ± 2.32 abcd	4.36 ± 1.86 ab	5.79 ± 2.14 ab	7.07 ± 1.59 a	1.86 ± 1.49 a	0.14 ± 0.24 ab	5.21 ± 2.10 ab	7.14 ± 1.55 a	7.43 ± 1.24 a
Marrone di Marradi IGP **	MT	198	5.67 ± 1.94 abc	4.78 ± 1.30 ab	5.22 ± 1.72 ab	6.22 ± 1.12 abc	1.83 ± 1.12 a	0.44 ± 0.73 ab	6.17 ± 2.12 ab	6.33 ± 1.20 ab	6.00 ± 1.32 abcd
Marrone della Val di Susa **	MT	47	2.64 ± 1.25 cd	4.21 ± 1.78 b	4.57 ± 2.23 ab	5.21 ± 1.78 abc	2.07 ± 1.84 a	0.57 ± 0.79 ab	6.21 ± 2.06 ab	6.33 ± 1.60 ab	5.92 ± 2.08 abcd
Marrubia *	SC	215	7.00 ± 0.65 a	5.07 ± 1.64 ab	6.21 ± 1.38 a	7.00 ± 0.82 ab	1.71 ± 1.38 a	0.00 ± 0.00 b	6.14 ± 1.11 ab	7.57 ± 0.98 a	7.71 ± 0.76 a
Marsol ***	EH	5	3.50 ± 0.71 b	4.00 ± 1.70 ab	6.00 ± 0 a	4.20 ± 1.96 a	1.90 ± 1.75 a	0.90 ± 0.74 a	5.10 ± 1.29 a	5.80 ± 1.44 a	5.60 ± 1.64 a
Neirana della Val di Susa *	SC	218	5.90 ± 1.65 ab	5.56 ± 2.01 ab	5.06 ± 1.83 ab	6.00 ± 1.98 abc	2.11 ± 1.41 a	0.44 ± 0.71 ab	5.89 ± 1.70 ab	5.72 ± 1.16 ab	5.56 ± 1.20 abcd
Precoce Migoule ***	EH	258	4.50 ± 1.53 ab	5.93 ± 1.37 a	4.14 ± 1.97 ab	4.71 ± 1.73 a	1.64 ± 1.11 a	0.29 ± 0.57 ab	5.64 ± 2.43 a	6.50 ± 0.87 a	5.43 ± 1.37 a
Tarvisò *	SC	25	3.21 ± 1.22 bcd	5.43 ± 1.99 ab	3.57 ± 1.75 ab	5.14 ± 2.25 abc	1.50 ± 1.38 a	0.14 ± 0.38 ab	5.14 ± 2.73 ab	5.86 ± 1.68 ab	5.93 ± 1.59 abcd

Mean value and standard deviation (SD) of each sample is given (*n* = 3). Different letters (a,b,c,d) for each descriptor indicate the significant differences at *p* ≤ 0.01. * Sweet chestnut (SC). ** Marrone-type chestnut (MT). *** Euro-Japanese hybrid (EH).

**Table 4:** Chestnut phytochemical class profiles.

		Cinnamic Acids		Flavonols		Benzoic Acids		Catechins		Tannins		Monoterpenes		Vitamin C	
		Mean Value	SD	Mean Value	SD	Mean Value	SD	Mean Value	SD	Mean Value	SD	Mean Value	SD	Mean Value	SD
CV	ID	g·kg^−1^ DW		g·kg^−1^ DW		g·kg^−1^ DW		g·kg^−1^ DW		g·kg^−1^ DW		g·kg^−1^ DW		g·kg^−1^ DW	
Bouche de Bétizac ***	159	0.204	0.001	0.089	0.007	0.014	0.001	0.079	0.007	0.173	0.022	3.365	0.118	0.124	0.018
Bouche Rouge *	286	0.151	0.080	0.011	0.000	0.025	0.000	0.049	0.001	0.060	0.008	0.643	0.158	0.189	0.029
Brunette *	9	0.158	0.080	0.085	0.001	0.036	0.001	0.065	0.002	0.174	0.008	1.614	0.095	0.126	0.008
Canepina *	78	0.208	0.001	0.244	0.034	0.064	0.007	0.159	0.034	1.201	0.348	7.590	1.788	0.055	0.023
Contessa *	30	0.199	0.005	0.145	0.024	0.027	0.000	0.019	0.002	0.196	0.004	2.595	0.576	0.160	0.023
Gabiana *	45	0.137	0.010	0.173	0.011	0.066	0.001	0.063	0.013	0.170	0.014	2.589	0.051	0.142	0.065
Garrone Rosso *	39	0.066	0.001	0.083	0.008	0.018	0.001	0.100	0.005	0.148	0.011	1.039	0.172	0.022	0.008
Gentile *	186	0.066	0.000	0.112	0.009	0.019	0.006	0.078	0.007	0.205	0.013	1.207	0.073	0.154	0.011
Madonna *	1	0.117	0.079	0.247	0.043	0.023	0.000	0.076	0.009	0.224	0.028	1.783	0.051	0.025	0.008
Mansa *	390	0.100	0.079	0.005	0.001	0.014	0.001	0.019	0.002	0.026	0.002	0.457	0.045	0.019	0.002
Marrone di Castel del Rio **	130	0.198	0.014	0.163	0.008	0.021	0.001	0.078	0.017	0.232	0.016	1.581	0.054	0.100	0.016
Marrone di Marradi IGP **	198	0.206	0.001	0.030	0.005	0.021	0.001	0.077	0.002	0.036	0.003	1.199	0.193	0.002	0.002
Marrone della Val di Susa **	47	0.210	0.003	0.118	0.010	0.022	0.001	0.075	0.003	0.192	0.020	2.812	0.454	0.019	0.002
Marrubia *	215	0.200	0.004	0.171	0.008	0.026	0.001	0.080	0.003	0.105	0.020	1.124	0.083	0.017	0.010
Marsol ***	5	0.199	0.004	0.072	0.005	0.024	0.001	0.100	0.008	0.117	0.009	1.822	0.061	0.027	0.006
Neirana della Val di Susa *	218	0.155	0.001	0.039	0.001	0.012	0.009	0.023	0.012	0.211	0.017	1.526	0.068	0.047	0.017
Precoce Migoule ***	258	0.209	0.001	0.044	0.002	0.025	0.000	0.106	0.005	0.036	0.006	7.371	0.478	0.025	0.005
Tarvisò *	25	0.202	0.001	0.256	0.031	0.015	0.001	0.109	0.006	0.652	0.041	0.958	0.343	0.105	0.015

Mean value and standard deviation (SD) of each sample is given (*n* = 3). * Sweet chestnut (SC). ** Marrone-type chestnut (MT). *** Euro-Japanese hybrid (EH).

**Table 5:** Nutritional properties of analysed chestnut cultivars.

		Organic Acids		Sugars	
		Mean Value	SD	Mean Value	SD
CV	ID	g·kg^−1^ DW		g·kg^−1^ DW	
Bouche de Bétizac ***	159	4.08	0.32	32.61	4.10
Bouche Rouge *	286	3.34	0.48	10.64	2.33
Brunette *	9	7.97	0.25	48.79	9.68
Canepina *	78	4.41	0.37	114.80	10.63
Contessa *	30	6.00	0.15	18.36	2.92
Gabiana *	45	3.88	0.43	38.32	3.86
Garrone Rosso *	39	1.20	0.21	14.90	2.07
Gentile *	186	2.63	0.19	48.97	2.19
Madonna *	1	4.34	0.80	62.47	3.38
Mansa *	390	6.58	0.71	273.38	21.16
Marrone di Castel del Rio **	130	2.49	0.39	26.80	3.91
Marrone di Marradi IGP **	198	3.18	0.31	29.22	2.03
Marrone della Val di Susa **	47	1.30	0.15	14.26	1.76
Marrubia *	215	1.79	0.31	25.29	4.01
Marsol ***	5	5.01	0.45	29.07	9.82
Neirana della Val Susa *	218	3.22	0.21	20.00	2.67
Precoce Migoule ***	258	7.43	0.09	38.97	1.40
Tarvisò *	25	4.75	0.35	25.75	1.34

Mean value and standard deviation (SD) of each sample is given (*n* = 3). * Sweet chestnut (SC). ** Marrone-type chestnut (MT). *** Euro-Japanese hybrid (EH).

## Supplementary Materials

The following are available online at https://www.mdpi.com/2304-8158/9/8/1062/s1.
